# Impact of COVID-19 on the Mental Health of Medical Students in Portugal

**DOI:** 10.3390/jpm11100986

**Published:** 2021-09-30

**Authors:** Ricardo Campos, Vânia Pinto, Daniela Alves, Celina Pires Rosa, Henrique Pereira

**Affiliations:** 1 Personalized Health Care Unit of Belmonte, 6250-072 Belmonte, Portugal; RSCampos@arscentro.min-saude.pt (R.C.); alves_danielaf@hotmail.com (D.A.); cprosa@arscentro.min-saude.pt (C.P.R.); 2 Local Health Unit of Alto Minho, 4904-858 Viana do Castelo, Portugal; vaniampereirapinto@gmail.com; 3 Department of Psychology and Education, Faculty of Social and Human Sciences, University of Beira Interior, Pólo IV, 6200-209 Covilhã, Portugal; 4 Research Centre in Sports Sciences, Health Sciences and Human Development (CIDESD), 5001-801 Vila Real, Portugal

**Keywords:** COVID-19, fear, mental health, medical students

## Abstract

(1) Background: The purpose of this article is to assess the impact of the COVID-19 pandemic on the mental health of medical students in Portugal in the period after returning to face-to-face classes during the COVID-19 pandemic, in the 2020/2021 academic year. (2) Methods: We conducted an observational, descriptive, and cross-sectional study, between December 2020 and February 2021 with a representative sample of Portuguese medical students (*n* = 649), applying an anonymous questionnaire which was composed by a sociodemographic characterization, The Brief Symptoms Inventory–18, The Fear of COVID-19 Scale and the Negative Impact Assessment Scale. For statistical processing, Statistical Package for Social Sciences (SPSS ©) was used. (3) Results: 65.3% of participants said that self-perceived relevant anxiety symptoms, and around 10% said that they had a physical or a mental illness diagnosis. Significant differences (*p* < 0.05) were found for Fear of COVID-19, Somatization, Anxiety and Overall Mental Health, indicating that women, students from the 1st and last years of training had higher scores. Age, year of training, Fear of COVID-19 and Negative Impact of COVID-19 were significant predictors of overall mental health. (4) Conclusion: In our sample of Portuguese medical students, age, year of training, but mostly fear of COVID-19 and the negative impact of COVID-19 contributed to mental health symptoms.

## 1. Introduction

The Coronavirus Disease 2019 (COVID-19) pandemic has had a major impact on the world population, both in terms of its morbidity and mortality rates and as for its social and economic impact. Undoubtedly, the COVID-19 has become one of the most important concerns of populations worldwide. In addition to the problems directly caused by the virus, psychosomatic symptoms involving fear and anxiety about being infected and infecting others are potentially worrying, thus justifying studies on the incidence of mental health symptoms, in the general population, but also in subgroups of the population. Since the health sector was one of the most affected by this pandemic, in addition to the structural changes inherent to the containment of the pandemic, an overload of work among health professionals was widely observed [1].

Medical students as future health professionals are also being directly and indirectly affected by the COVID-19 pandemic. The anticipation of a painful future in terms of work-related conditions, hardened in part by the pandemic, as well as the adaptation to university programs to the pandemic itself, could be potential causes of anxiety and other mental health problems among medical students [2].

Assessing the mental health functioning of a sample of medical students from Portuguese Universities is of uttermost importance in the period we are going through, as it is a population that already has important potential sources of anxiety. A recent meta-analysis estimated that the global prevalence of anxiety among medical students was around 33.8%, well above the prevalence in the general population [3]. In a study carried out in 2014, involving 557 Portuguese medical students from six Portuguese medical schools, significant anxiety levels were registered in 25.5% of the sample [4], and some validated screening tools for anxiety, depression and somatization have already been validated in Portuguese language [5,6]. Studies in other countries also point to significant levels of depression and anxiety in medical students. For example, in Australia, a cross-sectional survey was conducted among students enrolled from June to August 2009 in four Australian medical schools and approximately 25% of students reported a history of depression [7]. Also in Australia, the Systematic Review prior to this study encompassing articles between January 1980 and May 2005 pointed to the high prevalence of depression and anxiety among medical students, with levels of overall psychological distress consistently higher than the general population and age-matched peers by the later years of training [8]. Some Asian studies have also highlighted high levels of depression and anxiety among these students [9,10,11].

Many studies have been carried out since the outbreak of the COVID-19 pandemic, to assess its impact on the mental health of medical students. For instance, a study recently conducted in Portugal, concluded that students who had started the academic year in the pandemic period had significantly higher levels of depression, anxiety, and stress when compared to those who had enrolled the study programs in the non-pandemic period [2].

In fact, various studies have consistently showed an increase in anxiety and stress symptoms associated with the COVID-19 pandemic among medical students [12,13,14,15,16,17,18,19,20,21,22,23,24] as well an increase in depressive symptom [15,17,21,22,24,25]. The factors attributable to the pandemic explaining anxiety and depression may be due to being afraid of getting the infection and the risk of the development of severe illness and complications [26]. On the other hand, some studies showed that living in urban areas, living with parents, having economic stability, having social support and being younger appeared to be protective factors for depressive symptoms [13,16,24]. Self-efficacy and self-esteem were also found to be significant factors for the mediation of emotional distress [14].

Gender, especially being female, was shown in several studies to be a risk factor for anxiety and stress [12,15,17,18,19,20,23,24,27,28,29] as well as depressive symptoms [17] during the COVID-19 pandemic. Females tend to be more predisposed to express their feelings and are more likely to experience different social expectations, pressures, and gender equality-related positions [23]. However, other studies did not confirm this trend [13,14,16,26].

The year of study the medical program also showed to be associated with anxiety and depression [18], specially first years [23,24,30,31] and last years [15] of enrollment. Nevertheless, some research did not demonstrate this correlation [12,13,14]. Also, some studies refer that the main fears of students related with COVID-19 pandemic involve the negative impact on their studies and academic delays [12,16,18], economic outcomes, effects on daily life [16], fear of getting infected or transmitting the disease to family members [18,32], social distancing, and lockdown [26].

Social support demonstrates negative correlation with levels of anxiety [16] and this is one of the most effective positive factors to prevent stress and mental health symptoms [26]. In fact, psychological interventions designed to improve social support, have been shown to reduce perceived stress and are positive coping mechanisms that may be effective to improve the mental health of medical students [33].

Hence, the main objective of our study is to assess the impact of the COVID-19 pandemic on the mental health of medical students in Portugal in the period after returning to face-to-face classes during the COVID-19 pandemic, in the 2020/2021 academic year. More specifically, we aimed at assessing the levels of fear of COVID-19, negative impact of COVID-19, and levels of psychological symptoms (anxiety, depression, and somatization) among the sample.

## 2. Materials and Methods

We conducted an observational, descriptive, and cross-sectional study, between December 2020 and February 2021, that consisted in surveying a convenience sample of medical students’ representative of the universe of medical students in Portugal.

### 2.1. Ethical Approval

Before the beginning of the application of the questionnaire, the study was submitted to evaluation by two ethics committees, the first being the Ethics Committee of the University of Beira Interior from Covilhã (Portugal), and later the Ethics Committee of NOVA Medical School in Lisbon (Portugal), both issued favorable assessment to commence the study. By emitting the following approval codes, respectively, CE-UBI-Pj-2020-083 and No. 07/2021/CEFCM.

### 2.2. Sample Selection

As inclusion criteria for the sample, we chose to add individuals aged 18 years or older, enrolled in a medical program from a Portuguese medical school, in the academic year of 2020/2021. We excluded students with interrupted enrollment and who could not read Portuguese.

The minimum sample size was calculated for a 95% confidence interval with a 5% margin of error for a population of 12,575 students enrolled in the medical course in 2020 (data from the Contemporary Portugal Database [34] and the General Directorate Higher Education), having obtained the value of 373 individuals.

The sample was conveniently collected through online dissemination of the anonymous questionnaire. The dissemination was made through medical schools via institutional e-mail and by student associations from the same institutions, from December 2020 to February 2021. The anonymity of the respondents was guaranteed by hiding the Internet Protocol address of each participant’s servers, and by not including any identifying elements of the respondent in the questionnaire.

### 2.3. Questionnaire Protocol

The online questionnaire presented a brief description of the study so that the participant could consent to participate in the study. After authorized consent, the questionnaire was divided into several other sections:

1. a sociodemographic characterization of the sample
2. the Brief Symptoms Inventory-18
3. the Fear of COVID-19 scale

and the Negative Impact Assessment Scale.

The entire questionnaire was written in Portuguese, using the valid Portuguese versions of these scales.

#### 2.3.1. Brief Symptom Inventory-18

The Brief Symptom Inventory-18 (BSI-18) is a tool for screening psychological symptoms, consisting of 18 items grouped into three subscales (Somatization, Depression and Anxiety), each encompassing six items. Some items include: “feeling weak”, “faintness”, “feeling no interest in things”, “feeling hopeless about future”, “feeling tense” and “nervousness”. With the sum of the 18 items, Global Severity Index (IGG) can be obtained, which reflects the general level of psychological malaise of the individual. The answer options for each item are graded (from 0—Nothing to 4—Extremely). The BSI-18 is validated only for the adult population (over 18 years old). The BSI-18 was developed by Derogatis from two longer inventories developed by the same author: the Brief Symptom Inventory (BSI, with 53 items) and the Symptom Checklist-90-Revised (SCL-90-R, with 90 items), which assess nine dimensions of psychopathological symptoms: somatization, obsession-compulsions, interpersonal sensitivity, depression, anxiety, hostility, phobic anxiety, paranoid ideation and psychoticism. Subsequent studies have shown that BSI-18 is as effective as the longer questionnaires (BSI and SCL-90-R) in assessing psychopathological symptoms [6].

#### 2.3.2. Fear of COVID-19 Scale (FCV-19S)

Several studies have appeared in recent months and some scales have been vali-dated to measure anxiety and fear of COVID-19. The Fear of COVID-19 scale (FCV-19S), validated to assess COVID-19’s fear in general population [35], being used even in studies with samples of university students [36], some of them including medical students [37].

The FCV-19S, consists of 7 items in a 5-point Likert response (from 1—completely disagree; to 5 totally agree). The scale has recently been vali-dated in Portuguese [38]. The minimum score possible for each question is 1, and the maximum is 5. A total score is calculated by adding up each item score (ranging from 7 to 35). The higher the score, the greater is the fear of COVID-19.

Some examples of the items are: “I’m very afraid of COVID-19”, “I’m afraid of dying from COVID-19” or “I can’t sleep because I’m worried about getting COVID-19” [35].

#### 2.3.3. Negative Impact Assessment Scale

This new scale measures how negative the impact is perceived vis-à-vis COVID-19 compared to normal life before the pandemic. It consists of ten items with a five-point Likert-type response format (1 = nothing–5 = very much) and covers areas of psychosocial functionality, currently valid for the Portuguese population.

The scale covers areas of psychosocial functionality, such as: “Compared to my life before the COVID-19 pandemic, it had a negative impact on … my professional or academic life, … my relational life (relationships, friendships, etc.), … in my mental health, … in my financial life” [38].

### 2.4. Data Analyses

For statistical processing, the Statistical Package for Social Sciences (SPSS ©) was used.

Sociodemographic characteristics were described in absolute and relative numbers, using frequencies, percentages, mean, and standard-deviation.

For comparisons between two groups (genders), we used the T-Student, and for comparison of more than 6 groups (year of training), we used ANOVAs. To evaluate the association level between the variables, Pearson correlation coefficients were conducted. To determine possible predictive relationships, we conducted a hierarchical linear regression.

We computed the means of the 3 subscales of the BSI-18 into a general measure which we called Overall Symptoms, as it aims to encompass the 3 dimensions of the scale in a single measure.

## 3. Results

Table 1 shows sociodemographic characteristics of participants. The majority were female (78.4%), single 94.5%, lived in urban areas (78%), and attended the University of Lisbon (41.4%) and the University of Beira interior (32.2%); 65.3% said that self-perceived anxiety symptoms, and around 10% say that they had a physical or a mental illness diagnosis.

Table 2 shows results for overall scores for Fear of COVID-19, Negative Impact of COVID-19 and mental health variables. Moderate scores were found for all variables, when compared to community samples the mean of the sample responses on the Fear of COVID-19 scale is lower in our sample (2.18 vs. 3.91) [35], when we talk about the results of the overall mean of the Negative Impact of COVID-19 scale in our sample, the value is very close to the value of the validation study sample (2.69 vs. 2.60) [38]. The means of the Depression and Anxiety subscales of the BSI-18 were higher than those found in the Portuguese samples (respectively 1.06 vs. 0.89 and 1.24 vs. 0.82) as the mean value obtained by the sample in the subscale of somatization was slightly lower (0.55 vs. 0.57) [39].

Table 3 shows results for all variables by gender. Significant differences (*p* < 0.05) were found for Fear of COVID-19, Somatization, Anxiety and Overall Mental Health, indicating that women have higher scores in these variables.

Table 4 shows results for all variables by year of training. Significant differences (*p* < 0.05) were found for fear of COVID-19, Somatization, Depression, Anxiety and Overall Mental Health, indicating that 1st and last years of training scored higher.

A correlation matrix was conducted to assess association levels between variables under study. Significant associations were found (*p* < 0.05) for all variables. Overall Mental Health negatively correlated with age and year of training, and positively correlated with Fear of COVID-19 and Negative Impact of COVID-19 (Table 5).

Finally, a hierarchical linear regression analysis was conducted to determine the predictive effect of independent variables on Overall Mental Health. In the first block (Model I) possible confounding variables “age”, “gender”, and “year of training” were added, explaining 7% of overall variance. In the second block (Model II), Fear of COVID-19 and Negative Impact of COVID-19 were added, explaining 26% of overall variance. Age, year of training, Fear of COVID-19 and Negative Impact of COVID-19 were significant predictors of overall mental health (Table 6).

## 4. Discussion

In carrying out this study, we intended to assess the impact of the COVID-19 pandemic on the mental health of medical students in Portugal in the period after their return to face-to-face classes, evaluating the fear of COVID-19, the negative impact of COVID-19, and levels of psychological symptoms (anxiety, depression, and somatization) in our sample.

As main results of the study we highlight: (1) The sample mean in the FCV-19S is lower than in the scale validation sample [35]; (2) Means of the Depression and Anxiety subscales of the BSI-18 were higher than those found in the Portuguese samples [39]; (3) With regard to gender, females scored higher on Fear of COVID-19, Somatization, Anxiety and Overall Mental Health. (4) In the training year, the first and last years of enrollment scored higher in the Fear of COVID-19, Somatization, Depression, Anxiety and Overall Mental Health. (5) Overall Mental Health was negatively correlated with age and year of training, and positively correlated with Fear of COVID-19 and the Negative Impact of COVID-19. (6) Age, gender, year of training, Fear of COVID-19 and Negative Impact of COVID-19 were significant predictors of Overall Mental Health.

### 4.1. Overall Results for All Variables under Study

Regarding the global mean of the application of the FCV-19S scale, our sample registered a value of 2.18, which was much lower than the value of the sample in the scale validation study (3.91) [35]. This value indicates that our sample experienced, on average, fewer feelings of fear regarding the topic of COVID-19 than the sample in the validation study. The scale validation study was carried out at the beginning of the pandemic, when information about the virus and the evolution of the pandemic were scarcer, perhaps the lack of knowledge and the novelty of the COVID-19 theme could explain the higher levels of fear of this sample in relation to the sample in our study that was carried out several months after the beginning of the pandemic.

On the other hand, the proximity of the results between the global mean of the results in the Negative Impact of COVID-19 scale of our sample with the scale validation sample could potentially be due to the proximity of the application of the scale in temporal terms [38]. These results indicate that the negative impact of COVID-19 is similar between the two Portuguese samples, with both samples on average having scores higher than the scale’s average (which quotes responses from 0 to 5).

The means of the Depression and Anxiety subscales of the BSI-18 were higher than those found in the Portuguese samples (respectively 1.06 vs. 0.89 and 1.24 vs. 0.82) as the mean value obtained by the sample in the subscale of somatization was slightly lower (0.55 vs. 0.57) [39]. The fact that our sample consisted of medicine students and not a more diverse population may have contributed to this result. Portuguese medical students recorded levels of anxiety and depressive symptoms higher in other studies than university students from other courses. [3,40].

Anxiety symptoms experienced by medical students during the COVID-19 pandemic may also be explained by the impact of the virus on their studies and future employment, fear of being infected, forced distance from other people, and the lack of interpersonal communication. In a study conducted in China, social support demonstrated negative correlation with levels of anxiety, suggesting that the mental health of medical students should be a priority through measures evolving effective social support [16].

### 4.2. Comparison of Results by Genders

When we compared the two genders statistically significant differences in the Somatization and Anxiety subscales of the BSI-18 were found. In all of them, mean scores were higher among women (2.27 vs. 1.86 in FCV-19S and 1.01 vs. 0.39 and 1.29 vs. 1.01 respectively in somatization and anxiety-mind subscales). A study published in the pre-pandemic period found similar results between genders regarding the results of the 3 subscales of the BSI-18 [41].

Several other studies have pointed to a significant difference between genders in terms of anxiety, pointing to higher levels of anxiety and stress in females just like in our sample [12,15,17,18,19,20,23,24,27,28,29,37]. However, we also found some studies that did not observe statistically significant differences between genders in terms of anxiety and stress in their samples [13,14,16]. This shows the complexity associated with how gender may affect mental health functioning, as recent evidence suggests that biological factors, such as the variation in ovarian hormone levels, and psychosocial variables, such as social expectations, maybe associated with the increased prevalence of depression and anxiety in women [17,19,24,28].

In the case of the global means of the application of the BSI-18 depression subscale, the Negative Impact Assessment Scale and the Overall Symptoms measure, there was no statistically significant difference between both sexes.

### 4.3. Comparison of Results by Year of Training

When comparing the different years of medical training, we found that there was no statistically significant difference between the mean values obtained in the Negative Impact of COVID-19 scale. Due to being a recent scale, we could not find any study that applied it to a sample of medical students to compare the results with our sample.

When applying the FCV-19S scale, we found values that indicate a trend in the first two years of training towards higher levels of fear of COVID-19; on the other hand, more advanced years of training scored lower on the FCV-19S scale. Factors other than the year of training may be involved in the individual variability of the responses obtained. We found similar results in another study [37] indicating that, possibly, the fact that having more medical information and knowledge about the topic COVID-19 in latest years of training is associated with lower scores on the FCV-19S scale, because it allows students to perceive more control over the pandemic.

Regarding the three BSI-18 subscales, statistically significant differences were found by year of medical training. The first two years of training presented higher levels of somatiform symptomatology in relation to the others. We did not find scientific studies that evaluated somatic symptoms in samples of medical students using the BSI-18 subscale, to be able to compare with our sample.

Anxiety symptoms were also higher in the first year of training among our sample and decreased in the fourth and fifth years of training. We found several studies that had statistically significant differences between year of training for anxiety symptoms, even though the years of training with the highest levels of anxiety differed from study to study [18,23,24,42,43,44,45,46].

We also found statistically significant differences in our sample with regard to depressive symptoms across different training years. The first and sixth years experienced more depressive symptoms than the other four years of training. We did not find recent studies that applied the BSI-18 to medical students, however we found two studies that applied other scales and that also found significant differences between depressive symptoms in different years of training [15,24].

A possible explanation for these differences may be that the first year of training presents a greater challenge for students because it requires an adaptation to the university education, and, on the other hand, the sixth and final year of training presents greater pressure on the students for being the last and requiring an additional effort to complete the university studies and prepare the future health professional for the job market.

As expected from the individual results of the three BSI-18 subscales, the Overall Symptoms measure also had means with statistically significant differences between the different years of training. As in most subscales, the highest mean was for the first year of training, pointing to a greater tendency of these individuals to have symptoms related to mental health (depressive, anxious and somatic symptoms).

### 4.4. Correlation between Variables

Using Pearson’s Correlation Coefficient, we evaluated the correlation between eight variables, namely age, year of training, FCV-19S scores, somatization, anxiety, depression, and overall symptoms. We considered Evans [47] suggestion for the absolute value of *r*, and looking at their association with overall mental health symptoms, despite the fact that all were significant, year of training was very weakly associated, age and FCV-19S were weakly associated, while negative impact of COVID-19 was moderately associated. Somatization, anxiety, and depression were very strongly associated, which was expected since these are subscales belonging to the overall symptoms’ variable.

Factors associated with higher age and higher year of training were associated with lower FCV-19S and lower mental health symptoms. Other studies have also found similar evidence [43,44,45,46], and this may be related to the fact that younger and students of first years of training are still adapting to the university demands, exhibiting less productive coping mechanisms, and exposing higher levels of stress. On the other hand, older students may be already adapted to the university life and possess more scientific knowledge regarding specific pathologies (including COVID-19) which allows them to be in control, thus, exhibiting lower fear and higher mental health functioning.

Negative impact of COVID-19 was moderately associated with mental health symptoms, demonstrating that the pandemic compromises medical students. As medical schools can be seen as high-pressure environments, students are not immune to the added stressors presented by a global pandemic. Previous studies have shown that medical students are a population vulnerable to higher rates of mental health issues including somatization, anxiety, and depression [40,41,42].

### 4.5. Predictors of Overall Mental Symptoms

The comparison between the various dimensions that acted as predictors of overall mental health symptoms showed that age, and year of training, but mostly fear of COVID-19 and the negative impact of COVID-19 contributed to the explanation of the mental health symptoms. The fact that the pandemic has had an impact on the health system, politics, the economy, and education can be reflected in the appearance of several mental disorders, namely depression. Freshmen and younger students may have seen greater negative impact from COVID-19 and had higher levels of mental health symptoms and this level of concern and fear can be highly disabling. This is in line with other studies that have also observed similar results [37,40,41,42,43,44,46], indicating that medical students experienced significant impacts due to the stress added by the pandemic (here measured by the fear of COVID-19 and negative impacts of COVID-19) and have high levels of somatization, anxiety, and depression at the time of data collection.

Knowing beforehand that being a younger medical student and having more fear and higher negative impact of the pandemic in their liver, are significant predictors of poorer mental health functioning, students across these groups would benefit from tailored interventions aiming at minimizing the risk of psychological impact, not only because the pandemic remains ongoing, but especially to prevent future cost should future pandemic arise.

### 4.6. Study Limitations

The sample selection method (online questionnaire) may have unintentionally selected a sample more predisposed to participate, and therefore, impedes generalization of results. Possibly, most anxious participants were the most interested in the topic of COVID-19 and related subjects.

Another limitation has to do with our sample being disproportionately represented regarding genders, even though the Portuguese medical students’ population is also disproportionate (21.6% of male students in the sample vs. 31.3% in the Portuguese population) [34]. Also, the fact that we didn’t assess actual psychiatric diagnosis may come as a limitation, since this would require clinical data from individuals to confirm a supposed diagnosis.

When selecting the sample, we excluded students who could not read Portuguese, but we did not exclude students with non-Portuguese nationality enrolled in medicine programs in Portugal, and who were able to answer the questionnaire written in Portuguese. However, the questionnaire did not ask the participant’s nationality. It would have been interesting to have this information to compare results between subgroups. Future studies should address this limitation. We also excluded students who had interrupted their enrollment, so we may have excluded individuals with a potential propensity for psychological symptoms, as some of the interruptions may be due to psychiatric pathology. It would have been interesting to include this subgroup of students (although it is a residual group) and inquire them about the reasons for interrupting and try to compare their results with other groups in the sample.

When discussing our results, different scales were used to evaluate psychological symptoms among different studies, which may have affected the comparison of data and lead to variance between findings. On the other hand, training years in medical school are different among the countries, and this prevents generalization of results among other nations.

## 5. Conclusions

The COVID-19 pandemic has had an impact on the mental health of medical students around the world. Our study shows that the mental health needs of Portuguese medical students during the COVID-19 pandemic should be addressed, and interventions by the educational authorities and medical schools are necessary. Psychological well-being of medical students should be a major concern given their future responsibilities, and a priority when designing educational programs.

Our study provides feedback to medical schools on the impact of COVID-19 on students’ mental health. As this can be detrimental to their future health, as well as to their learning, it can be useful to create psychological support offices in educational institutions to help students develop resilience and coping strategies in relation to adversities, which may also have an impact in their future responsibilities as medical professionals.

Previous research shows negative correlation between self-efficacy and anxiety, suggesting measures to increase self-efficacy as cognitive-behavioral therapy and mindfulness-based interventions [14]. The subgroups of female students, due to their greater propensity to anxiety and somatic symptoms, should also be a source of particular attention by universities in the planning of psychological support, as they potentially constitute a vulnerable group in this regard. The same can be used for some phases of the academic trajectory of these students, namely the first and last years of training, where the Portuguese Medicine student also seems to be more vulnerable to psychosocial symptoms.

We suggest carrying out more studies that explore possible causes for these differences between gender and years of training. In these studies, they may include more variables to obtain more subgroups that can be studied and compared with each other. We suggest including nationality, socioeconomic status, and the existence of previous psychiatric pathology as variables to be studied in more detail in future studies. We also suggest future research to include surveys of possible stressors that can be correlated with the remaining variables and include the issue of substance-related addiction and substance-free addiction as possible coping mechanisms. Follow-up surveys would be relevant to understand the possible later onset of mental health disease.

## Figures and Tables

**Table 1:** Sociodemographic Characteristics (*n* = 649; *M_age_* = 22.45; *SD* = 4.08).

Variable	Categories	*n*	%
Gender	Women	509	78.4
	Men	140	21.6
Marital Status	Single	623	94.5
	Married	19	2.9
	De facto union	16	2.5
	Divorced	1	0.2
Place of residence	Small rural	82	12.6
	Big rural	73	11.2
	Small urban	253	39
	Big urban	240	37
Socioeconomic status	Low	6	0.9
	Low-medium	72	11.1
	Medium	407	62.7
	Medium-high	157	24.2
	High	7	1.1
Medical School attended	University of Minho	72	11.1
	Medical School of University of Porto (FMUP)	2	0.3
	Institute of Biomedical Sciences University of Porto (ICBAS)	35	5.4
	University of Beira Interior (FCS-UBI)	209	32.2
	University of Coimbra (FMUC)	2	0.3
	University of Lisbon (FMUL)	269	41.4
	NOVA Medical School	34	5.2
	University of Algarve	22	3.4
	University of Madeira	4	0.6
Year of training	1st year	114	17.6
	2nd year	114	17.6
	3rd year	94	14.5
	4th year	95	14.6
	5th year	105	16.2
	6th year	127	19.6
Attendency of hospital internship	Yes	417	64.3
	No	232	35.7
Self-perceived anxiety symptoms	Yes	424	65.3
	No	225	34.7
Self-identified problems in life	Medical School-related	256	39.4
	COVID-19-related	74	11.4
	Family-related	42	6.5
	Physical illness	3	0.5
	Mental illness	30	4.6
	Other	17	2.6
Physical illness diagnosed	Yes	71	10.9
	No	578	89.1
Mental illness diagnosed	Yes	66	10.2
	No	583	89.8

Note: *n*—sample size; %—percentage.

**Table 2:** Overall results for all variables under study.

	*M*	*SD*
Fear of COVID-19	2.18	0.71
Negative Impact of COVID-19	2.69	0.75
Somatization Symptoms	0.55	0.59
Depressive Symptoms	1.06	0.82
Anxiety Symptoms	1.24	0.82
Overall Symptoms	0.95	0.66

Note: *M*—mean; *SD*—standard deviation.

**Table 3:** Results for all variables by gender.

		*M*	*SD*	*t (df)*	*p*
Fear of COVID-19	Women	2.27	0.71	6.264 (647)	0.000 **
	Men	1.86	0.62		
Negative impact of COVID-19	Women	2.70	0.71	0.205 (647)	0.837
	Men	2.68	0.62		
Somatization	Women	1.01	0.61	3.384 (647)	0.001 *
	Men	0.39	0.50		
Depression	Women	1.01	0.61	0.737 (647)	0.462
	Men	0.39	0.49		
Anxiety	Women	1.29	0.61	3.283 (647)	0.001 *
	Men	1.01	0.70		
Overall Symptoms	Women	0.98	0.67	2.687 (647)	0.007 *
	Men	0.81	0.55		

Note: *M*—mean; *SD*—standard deviation; *t(df)*—*t* student (degrees of freedom); *p*—probability value, * <0.05; ** <0.001.

**Table 4:** Results for all variables by year of training.

		*M*	*SD*	*F*	*p*
Fear of COVID-19	1st	2.30	0.70	2.769	0.017 *
	2nd	2.33	0.77		
	3rd	2.11	0.69		
	4th	2.13	0.70		
	5th	2.07	0.73		
	6th	2.10	0.64		
Negative impact of COVID-19	1st	0.72	0.06	0.105	0.991
	2nd	0.76	0.07		
	3rd	0.80	0.08		
	4th	0.80	0.08		
	5th	0.73	0.07		
	6th	0.72	0.06		
Somatization	1st	0.66	0.06	4.538	0.000 **
	2nd	0.63	0.05		
	3rd	0.56	0.05		
	4th	0.52	0.05		
	5th	0.57	0.05		
	6th	0.50	0.04		
Depression	1st	0.87	0.08	4.549	0.000 **
	2nd	0.80	0.07		
	3rd	0.81	0.08		
	4th	0.74	0.07		
	5th	0.77	0.07		
	6th	0.84	0.07		
Anxiety	1st	0.88	0.08	3.654	0.003 *
	2nd	0.82	0.07		
	3rd	0.82	0.08		
	4th	0.75	0.07		
	5th	0.75	0.07		
	6th	0.82	0.07		
Overall Symptoms	1st	0.71	0.06	5.120	0.000 **
	2nd	0.64	0.06		
	3rd	0.65	0.06		
	4th	0.58	0.06		
	5th	0.61	0.06		
	6th	0.64	0.05		

Note: *M*—mean; *SD*—standard deviation; *F*—ANOVA; *p*—probability value, * <0.05, ** <0.001.

**Table 5:** Correlation Matrix.

	1	2	3	4	5	6	7	8
1—Age	1							
2—Year of training	0.475 **	1						
3—Fear of COVID-19	−0.128 **	−0.124 **	1					
4—Negative Impact of COVID-19	−0.083 *	−0.010	0.351 **	1				
5—Somatization	−0.136 **	−0.180 **	0.329 **	0.350 **	1			
6—Depression	−0.223 **	−0.153 **	0.228 **	0.375 **	0.545 **	1		
7—Anxiety	−0.199 **	−0.150 **	0.380 **	0.384 **	0.697 **	0.717 **	1	
8—Overall Symptoms	−0.217 **	−0.181 **	0.352 **	0.422 **	0.819 **	0.881 **	0.926 **	1

* <0.05; ** <0.001.

**Table 6:** Hierarchical linear regression analysis predicting Mental Health.

	Model I			Model II		
	*B*	*SE B*	*β*	*B*	*SE B*	*β*
Age	−0.028	0.007	−0.171 **	−0.019	0.006	−0.118 *
Gender	−0.150	0.061	−0.094 *	−0.073	0.056	−0.046
Year of training	−0.034	0.016	−0.092 *	−0.034	0.014	−0.093 *
Fear of COVID-19				0.179	0.035	0.194 **
Negative Impact of COVID-19				0.299	0.032	0.343 **
*R^2^*	0.065			0.255		
*F*	14.599 **			45.315 **		

Note: *B*—unstandardized beta; *SE B*—standard error for unstandardized beta; *β*—*t* test statistic; *p*—probability value; *R*^2^—Coefficient of Determination; *F*—value obtained from a regression analysis to compare means of two populations, * < 0.05, ** <0.001.

## Data Availability

The data presented in this study are available upon request.
